# Cytomegalovirus proctitis as a complication of COVID-19 with immunosuppressive treatments

**DOI:** 10.1016/j.idcr.2021.e01111

**Published:** 2021-04-05

**Authors:** François Maillet, Annabelle Pourbaix, Diane le Pluart, Laura Sirmai, Speranta Andreea Postolache, Anne Couvelard, Nadhira Houhou-Fidouh, Lisa Males, Laurène Deconinck, François-Xavier Lescure

**Affiliations:** a Infectious Diseases Department, AP-HP Bichat Claude-Bernard Hospital, 75018, Paris, France; b Hepatogastroenterology Department, AP-HP Bichat Claude-Bernard Hospital, 75018, Paris, France; c Pathology Department, AP-HP Bichat Claude-Bernard Hospital, 75018, Paris, France; d Virology Department, AP-HP Bichat Claude-Bernard Hospital, 75018, Paris, France; e Radiology Department, AP-HP Bichat Claude-Bernard Hospital, 75018, Paris, France

**Keywords:** COVID-19, Cytomegalovirus infections, Rectal diseases, Proctitis, Dexamethasone, Anakinra

## Abstract

We report a case of reactivated biopsy-proven cytomegalovirus proctitis complicating the course of severe COVID-19 pneumonia treated with dexamethasone, anakinra and lopinavir/ritonavir. No other contributing factor was found than iatrogenic immunosuppression and COVID-19 immune dysregulation. We draw attention to the immunosuppressive risk when treating severe COVID-19 pneumonia with immunomodulators.

## Introduction

Cytomegalovirus (CMV) is a DNA virus of the *Herpesviridae* family, which infection is very common and mostly asymptomatic in immunocompetent hosts. Nevertheless, CMV disease can develop as a complication of CMV reactivation during an immunosuppressed state. Although CMV disease is well described in immunosuppressed patients such as HIV-infected patients or solid organ transplant recipients, we describe the case of a prior immunocompetent man who developed CMV reactivation with proctitis following severe SARS-CoV-2 infection needing immunosuppressive treatments.

## Case report

A 75-year old man presented to our emergency department for hypoxemic respiratory distress, with a 15-day history of fever, chills, myalgias and cough. His medical history consisted in uncomplicated well-controlled type 2 diabetes and overweight (BMI being 26.2 kg/m^2^).

A 6 L/min oxygen therapy was initiated and the patient was diagnosed with severe COVID-19 pneumonia: the RT-PCR testing for SARS-CoV-2 from nasopharyngeal swab was positive, and the chest computed tomography (CT) confirmed a typical radiologic pattern with a 50 % lung parenchymal involvement. Routine blood tests showed an inflammatory syndrome with elevated CRP (100 mg/L) and neutrophilia (11,000/μL), with global lymphopenia (700/μL), but without liver or renal function abnormalities. He was treated with a 10-day course of intravenous (IV) dexamethasone (20 mg/day during 5 days, followed by 10 mg/day during 5 days) and with a 7-day course of ceftriaxone and spiramycin for suspected bacterial super-infection.

After 4 days of dexamethasone and antimicrobial treatment, his medical condition worsened: his oxygen therapy level was 15 L/min, blood inflammatory syndrome increased and a new CT scan showed a worsening up to 60 % of parenchymal involvement. A combined treatment with a 10-day course of antiviral therapy (oral lopinavir/ritonavir, 400 mg twice a day) and a 5-day course of anti-interleukin-1 receptor antagonist (subcutaneous anakinra, 100 mg/day) was added to corticosteroids. His respiratory state progressively improved with apyrexia and decrease of oxygen requirement.

**Fig. 1:**
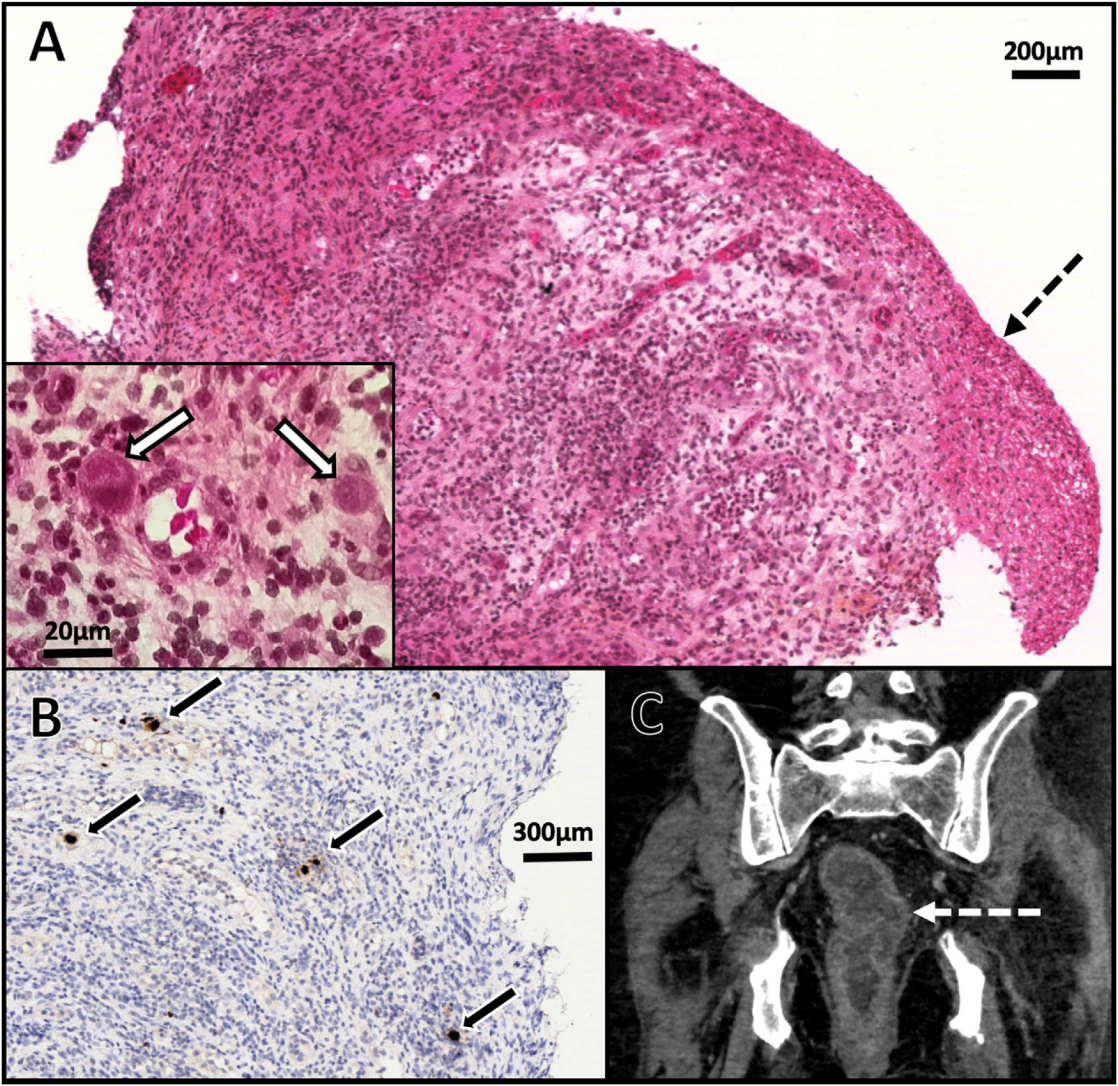
**Rectal biopsy and abdominal computed tomography.** A. Rectal mucosa biopsy, hematoxylin-eosin staining, optical microscopy. Mucosal injury with inflammation and ulceration (black dashed arrow) and many cytomegalovirus inclusion bearing cells. Insert shows ballooned cells with cytomegalovirus inclusions at higher magnification (white arrows). B. Rectal mucosa biopsy, immunohistochemistry with an anti-CMV antibody. Multiple cytomegalovirus infected cells in the ulcerated area (black arrows). C. Iodinated contrast agent-enhanced abdominal computed tomography, coronal section. Rectal wall thickening with mucosal edema and peri-rectal fat infiltration: uncomplicated proctitis.

One week after the start of anakinra, he presented fever and acute non-bloody diarrhea. An abdominal iodinated contrast agent-enhanced CT revealed a circumferential thickening of the rectal wall, with peri-rectal fat infiltration and enhancement of rectal mucosa, consistent with uncomplicated proctitis (Fig. 1). Repeated stool bacterial and parasitic cultures were negative, as well as a fecal multiplex PCR (DiagCORE® Gastrointestinal panel, Qiagen) and *Clostridioides difficile* infection studies. Diarrhea worsened, and he was transferred in intensive care unit for hypovolemic shock. A colonoscopy showed multiple uncomplicated diverticula in the sigmoid colon, and a circumferential mass of the lower and medial parts of the rectum, that was biopsied. Pathologic examination of the rectal biopsy (Fig. 1) revealed an ulcerated rectal mucosa and a cytopathogenic effect with ballooned cells (“owl’s eye”) and cytomegalovirus inclusions. Specific immunohistochemistry staining with an anti-CMV antibody was positive on rectal biopsy. Blood CMV specific PCR was positive with a 3.14 log UI/mL viral load (1848 copies/mL), and two serologies with a 15-day interval showed stable positive IgG with negative IgM. The diagnosis of reactivated CMV proctitis was made.

Our patient was treated with a 3-week course of 900 mg twice daily oral valganciclovir, with a favorable outcome. At the end of this treatment, his transit was back to normal and the fever had disappeared. An abdominal CT showed complete resolution of the proctitis, and serum CMV PCR was undetectable. As he was clinically cured and that all immunosuppressive agents had been stopped more than 2 months ago, he did not receive secondary prophylaxis.

We did not find any underlying chronic condition that could explain this reactivated CMV proctitis: HIV serology was negative, total blood lymphocyte count was 1600/μL with a CD4+ T-cell count of 710/μL (2 months after the beginning of COVID-19 pneumonia). He did not report any repeated infections that could reveal a primary or secondary immune deficiency, serum IgG, IgA and IgM levels were normal (1443 mg/dL, 208 mg/dL and 101 mg/dL, respectively), and serum lymphocyte phenotyping was normal. Inflammatory bowel disorders can also be complicated or revealed by a CMV proctitis. He therefore underwent a rectosigmoidoscopy two months after the end of valganciclovir treatment. Neither macroscopic aspect nor multiple biopsies did provide any clue for this hypothesis. Pathologic examination and immunohistochemistry were negative for CMV.

## Discussion

Gastro-intestinal (GI) symptoms, mostly diarrhea, nausea and abdominal pain, are very frequent in COVID-19, and occur in 33 % of patients [1]. Nevertheless, severe or prolonged GI symptoms should lead to an exhaustive research of other causes, including infectious diseases. Cytomegalovirus co-infection or reactivation seems to be able to complicate the course of COVID-19. Indeed, as of this publication, three other patients with severe COVID-19 pneumonia have been reported with CMV co-infection or reactivation-related GI symptoms [[2], [3], [4]]. We therefore believe that unusual GI symptoms in the settings of COVID-19 should lead to systematic GI exams, including both upper and lower digestive tract endoscopies with specific CMV examination, and a serum CMV PCR.

In this case, two associated factors contributed to CMV disease. First, dysregulation of immune system during COVID-19 pneumonia have been described, especially in severe cases [5,6]. Patients with COVID-19 and respiratory failure have a significantly decreased lymphocyte count, both globally and in all subsets. There is also a specific decrease in CD4+ T-cells, especially in memory and regulatory CD4+ T-cells subsets [6]. As in HIV patients, CD4+ lymphopenia, even transient, could lead to CMV disease. Other mechanisms, such as cytokine-mediated immune dysregulation or innate immune system disorganization, could be involved in the pathogenesis of relative immunosuppression during COVID-19 pneumonia.

Second, some immunosuppressive agents may be effective against the deleterious effects of COVID-19 inflammatory cytokine storm syndrome, but could also lead to potential infectious adverse events. Our patient was treated with both IV dexamethasone and subcutaneous anakinra. Dexamethasone is a highly immunosuppressive steroid which have been reported, in a large randomized controlled trial, to reduce 28-day mortality, hospital discharge and receipt of invasive mechanical ventilation or death in patients with moderate to severe COVID-19 pneumonia [7]. Its role in CMV disease is complex. Dexamethasone could favor CMV replication or disease, as laboratory models showed an enhanced CMV replication in epithelial cell cultures treated with dexamethasone compared to control cultures [8]. For example, CMV uveitis is a known adverse effect of intraocular dexamethasone implants, and have been described after systemic steroids use, even in prior non immunocompromised patients [9,10]. Anakinra, an anti-IL-1 receptor antagonist, has been reported in retrospective case series or case reports to be associated with a favorable outcome in patients with severe COVID-19 pneumonia, suggesting a higher survival rate, a decrease in the need of mechanical ventilation and in inflammatory markers in anakinra-treated patient [11,12]. Nevertheless, CMV disease has never been described as a potential side effect of anakinra. Interestingly, in the three previously reported patients with digestive CMV reactivation, all patients had been treated with immunosuppressive treatments. None of them had received anakinra and two had received steroids. This could indicate that CMV reactivation is not a specific side effect of these treatments, but a consequence of this second immunosuppressive hit following COVID-19 immune dysregulation.

Our case highlights the potential adverse effects of COVID-19 immunosuppressive agents. We believe that physicians should consider the immunosuppressive risk while treating severe COVID-19 pneumonia, as severe opportunistic infections, usually happening in very immunosuppressed patients, are increasingly reported.

## Author contribution

François Maillet, Annabelle Pourbaix, Diane le Pluart and Laurène Deconinck wrote the first draft of the manuscript.

François Maillet, Lisa Males, Speranta Andresse Postolache, Anne Couvelard, Nadhira, Houhou-Fidouh and Laura Sirmai collected the data.

François-Xavier Lescure corrected the final draft of the manuscript.

## Funding

This research did not receive any specific grant from funding agencies in the public, commercial, or not-for-profit sectors.

## Ethical approval

Written informed consent was obtained from the patient for publication of this case report and accompanying images. A copy of the written consent is available for review by the Editor-in-Chief of this journal on request.

## Patient consent

Written informed consent was obtained from the patient for publication of this case report and accompanying images. A copy of the written consent is available for review by the Editor-in-Chief of this journal on request.

## Declaration of Competing Interest

All authors declare they do not have any competing interest regarding this work.
